# Quantitative microbial risk assessment of *Campylobacter jejuni* in jerky in Korea

**DOI:** 10.5713/ajas.18.0322

**Published:** 2018-07-26

**Authors:** Jimyeong Ha, Heeyoung Lee, Sejeong Kim, Jeeyeon Lee, Soomin Lee, Yukyung Choi, Hyemin Oh, Yohan Yoon

**Affiliations:** 1 Department of Food and Nutrition, Sookmyung Women’s University, Seoul 04310, Korea; 2 Risk Analysis Research Center, Sookmyung Women’s University, Seoul 04310, Korea

**Keywords:** *Campylobacter jejuni*, Jerky, Quantitative Microbial Risk Assessment

## Abstract

**Objective:**

The objective of this study was to estimate the risk of *Campylobacter jejuni* (*C. jejuni*) infection from various jerky products in Korea.

**Methods:**

For the exposure assessment, the prevalence and predictive models of *C. jejuni* in the jerky and the temperature and time of the distribution and storage were investigated. In addition, the consumption amounts and frequencies of the products were also investigated. The data for *C. jejuni* for the prevalence, distribution temperature, distribution time, consumption amount, and consumption frequency were fitted with the @RISK fitting program to obtain appropriate probabilistic distributions. Subsequently, the dose-response models for *Campylobacter* were researched in the literature. Eventually, the distributions, predictive model, and dose-response model were used to make a simulation model with @RISK to estimate the risk of *C. jejuni* foodborne illness from the intake of jerky.

**Results:**

Among 275 jerky samples, there were no *C. jejuni* positive samples, and thus, the initial contamination level was statistically predicted with the RiskUniform distribution [RiskUniform (−2, 0.48)]. To describe the changes in the *C. jejuni* cell counts during distribution and storage, the developed predictive models with the Weibull model (primary model) and polynomial model (secondary model) were utilized. The appropriate probabilistic distribution was the BetaGeneral distribution, and it showed that the average jerky consumption was 51.83 g/d with a frequency of 0.61%. The developed simulation model from this data series and the dose-response model (Beta Poisson model) showed that the risk of *C. jejuni* foodborne illness per day per person from jerky consumption was 1.56×10^−12^.

**Conclusion:**

This result suggests that the risk of *C. jejuni* in jerky could be considered low in Korea.

## INTRODUCTION

In recent years, the meats used to produce jerky have expanded to beef, pork, and chicken [1,2]. Jerky is a nutritious meat product, and it has a long shelf life, because of its low water activity [3]. However, foodborne outbreaks of microbial diseases have been reported in many countries [4–6]. In recent, *Campylobacter* foodborne outbreaks have been dramatically increased. This increase may occur by advanced detection methods than the past, rather than actual increase of the outbreak. *Campylobacter* outbreaks were obviously under-estimated because of inaccurate detection method. Thus, necessity of risk assessment for *Campylobacter* has been suggested.

*Campylobacter* species are Gram-negative, microaerophilic bacilli that have shaped like curved rods or spirals [7,8]. In the United States, most of the reported *Campylobacter* infections are caused by *Campylobacter jejuni* (*C. jejuni*) [9]. *C. jejuni* grows well in microaerophilic conditions, such as 5% O_2_, 10% CO_2_, and 85% N_2_ environments, and it is sensitive to drying, acidic conditions, and salinity [10]. Additionally, it is a normal intestinal flora of animals, such as cattle, sheep, and poultry [11,12]. *C. jejuni* is a common bacterium that causes acute gastroenteritis worldwide [7]. In general, the symptoms of *C. jejuni* infection are diarrhea, fever, and abdominal cramps. Importantly, following an infection with *C. jejuni*, Guillain-Barre syndrome, which is an acute demyelinating disease of the peripheral nervous system, is possible [13].

Quantitative microbial risk assessment (QMRA) is widely used as a tool to describe the microbial risk levels of foods [14]. The QMRA consists of hazard identification, exposure assessment, hazard characterization, and risk characterization [15]. In hazard identification, the adverse effects on public health are defined, and the exposure assessment estimates the quantitative hazards in food at the point of consumption [16]. Dose-response models are used in hazard characterization to calculate the probability of foodborne illness [16], and in risk characterization, the risk is calculated from the exposure assessment and hazard characterization data [17]. The QMRA result is used to suggest quantitative criteria of foodborne pathogens, but many countries still do not have quantitative criteria for jerky [18,19]. Also, in Korea there are no quantitative criteria for *C. jejuni* in jerky. Therefore, the objective of this study was to evaluate the risk of *C. jejuni* foodborne illness from various jerkies in Korea.

## MATERIALS AND METHODS

### Prevalence level of *Campylobacter jejuni*

To evaluate the contamination levels of *C. jejuni*, seasoned (n = 125) and non-seasoned beef and poultry jerky (n = 150) were purchased from conventional markets, grocery stores, or online shops in Korea. Ten-gram portions of the samples were placed into sterile filter bags (3M, St. Paul, MN, USA), and 40 mL of Bolton broth (Oxoid Ltd., Basingstoke, UK), supplemented with 5% laked horse blood, was added and homogenized for 90 s. They were then incubated at 37°C for 4 h, followed by incubation at 42°C for 44 h. The incubated homogenates were then streaked onto modified charcoal-cefoperazone-deoxycholate agar (mCCDA; Oxoid Ltd., UK) and incubated at 42°C for 48 h under microaerobic conditions (2.5% to 9.5% CO_2_, 6.2% to 13.2% O_2_) using CampyGen (CampyGen gas generating system, Oxoid Ltd., UK). The presumptive *C. jejuni* colony on the mCCDA was streaked onto two Colombia agar plates (bioMérieux, Marcy-l’Étoile, France), and one plate was incubated under aerobic conditions and the other one was incubated under microaerobic conditions at 42°C for 48 h. Further analysis, using PCR to identify *C. jejuni*, was conducted, when colonies were formed only on the plate that was placed in the microaerobic environment [20]. Additionally, the homogenates were plated onto mCCDA, and the plates were incubated at 42°C for 48 h under microaerobic conditions for quantitative analysis. Presumptive colonies were then counted, and five randomly selected colonies on each plate were analyzed by using of PCR to identify *C. jejuni*. To amplify the constant sequence, primers of C-1F (5′-CAAATAAAGTTAGAGGTA GAATGT-3′) and C-3R (5′-CCATAAGCACTAGCTAGCTG AT-3′) were used [21]. PCR amplification was performed with a 20 μL reaction volume using the Fastmix Frenche PCR Premix kit (iNtRON Biotechnology, Seongnam, Korea), 2 μL of each primer, 2 μL of template DNA, and 14 μL of distilled water. The amplification profile was an initial denaturation step at 95°C for 15 min, and at 95°C for 30 s, 58°C for 90 s, and 72°C for 60 s for annealing. The annealing step had 25 cycles. Subsequently there was a final extension step at 72°C for 7 min. To confirm the amplification of the target sequence, the PCR product was electrophoresed on a 1.5% agarose gel in 1×Tris-acetate-ethylenediaminetetraacetic acid buffer (Biosesang, Seongnam, Korea) at 100 V for 20 min. The positive ratio was multiplied by the number of colonies to estimate the *C. jejuni* counts. However, the *C. jejuni* counts were below the detection limit (0.48 log colony-forming unit [CFU]/g), and thus, the *C. jejuni* prevalence data were fit to a uniform distribution [RiskUniform (α: minimum value, β: maximum value)].

### Development of a predictive model

To describe the changes in the *C. jejuni* cell counts during distribution and storage, predictive models were developed. *C. jejuni* NCTC11168 was stored at −70°C in bead stock (AES Chemunex, Combourg, France). One of the beads was streaked on Columbia agar and incubated at 42°C for 48 h under microaerobic conditions. The colonies on the plates were collected by scraping with a loop, and they were again streaked on Columbia agar; the plates were then incubated for 48 h. The colonies were collected in 5 mL of phosphate-buffered saline (PBS; pH 7.4; 0.2 g of KH_2_PO_4_, 1.5 g of Na_2_HPO_4_·7H_2_O, 8.0 g of NaCl, and 0.2 g of KCl in 1 L of distilled water). The suspensions were centrifuged at 1,912×g for 15 min at 4°C and washed twice with PBS. Then, the supernatants were discarded, and the cell pellets were resuspended in PBS. The optical density measured at 600 nm of the suspension was adjusted to 2.0 (ca. 5.5 log CFU/mL) for the inoculum. Seasoned or non-seasoned beef jerky was purchased from an online shop in Korea. Ten-gram portions of the samples were placed into a sterile filter bag, and 0.1-mL portions of the inoculum were inoculated on the jerky surface in the sample bag. The samples were rubbed 20 times and packaged aerobically or anaerobically, followed by storage at 10°C, 20°C, 25°C, and 30°C. Jerky samples were analyzed at the appropriate time intervals. Then, 30 mL of 0.1% buffered peptone water (BPW; Becton, Dickinson and Company, Franklin Lakes, NJ, USA) was added to each sample, and they were homogenized with a BagMixer (Interscience, St. Nom, France) for 90 s. The homogenates were serially diluted with BPW. One-tenth of 1 mL of the diluents was plated on mCCDA for *C. jejuni*, and the plates were incubated microaerobically. The typical colonies on the plates were manually counted. Then, the Weibull model was fit to the *C. jejuni* cell count data [22];

$$\text{Log}(N)=\text{Log}(N_{0})-{\left(\text{time}/\delta \right)}^{\rho }$$

Where *N_0_* is the initial number of cells, ρ is the shape of curve, and δ is required time for the first decimal reduction. To evaluate the effect of the storage temperature on δ, a polynomial model was used. Additionally, to evaluate the model performance, *C. jejuni* cell count data were collected at 15°C and 23°C through additional experiments. These observed data were compared to the predicted data from the predictive model. The accuracy between the observed and predicted data was expressed as a value from the root mean square error (RMSE) [23];

$$\text{RMSE}=\sqrt{\frac{1}{n}\times \sum {\left(\text{observed data}-\text{predicticed data}\right)}^{2}}$$

Where n represents the number of data points.

### Storage temperature and jerky consumption in Korea

The market storage temperature was collected by personal communication in this research. The transportation temperature from the market to home was collected in a study by Jung [24]. For the daily consumption amount and frequency of jerky consumption, Ministry of Food and Drug Safety [25] data were used. These data were fitted to @RISK version 6.0 (Palisade Corp., Ithaca, NY, USA) to determine the appropriate probabilistic distributions.

### Hazard characteristics and risk characterization

To calculate the probability of foodborne illness, the dose-response model, developed by Teunis and Havelaar [26] was utilized. To calculate the probability of foodborne illness from *C. jejuni* through jerky consumption, a simulation model, which was a series of prevalence, contamination levels, storage temperature and time distribution, consumption amount and frequency, and dose-response model, was prepared in the @RISK program. Eventually, the risk of foodborne illness was calculated through 10,000 iterations of random sampling.

## RESULTS AND DISCUSSION

### Prevalence and initial contamination level of *Campylobacter jejuni*

The initial contamination level of *C. jejuni* on the seasoned and non-seasoned jerky was investigated, and *C. jejuni* was below the detection limit in all of the samples (n = 275). It was assumed that the contamination levels were distributed between 0 CFU/g and below the detection limit, and thus, a uniform distribution [RiskUniform (−2, 0.48)] was determined to be appropriate to describe the distribution of the *C. jejuni* contamination levels. The parameters indicate that the *C. jejuni* contamination levels are distributed between −2.0 and 0.48 log CFU/g.

### Predictive model

**Figure 1:**
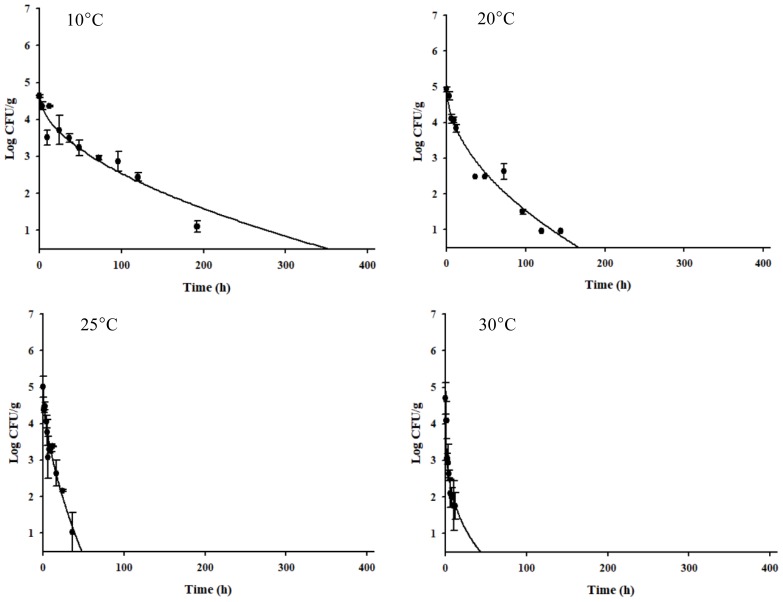
Cell counts of *Campylobacter jejuni* in seasoned jerky products during vacuum storage at 10°C, 20°C, 25°C, and 30°C. Symbol, observed cell counts; line, line fit with the Weibull model [22].

**Figure 2:**
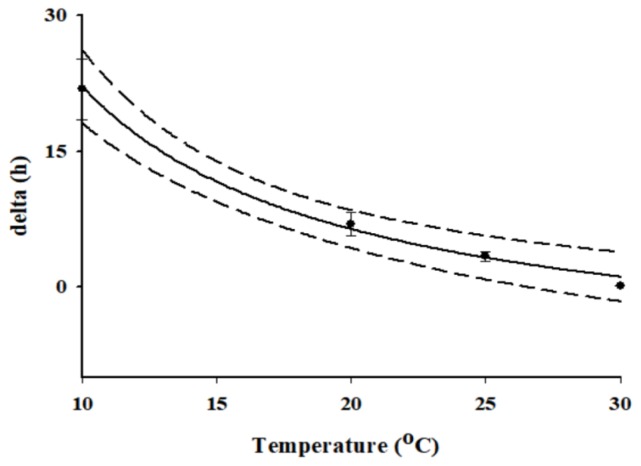
Observed δ values from the primary model and the fitted lines by a secondary model that describe the effect of temperature on δ for *Campylobacter jejuni* in seasoned jerky products.

The *C. jejuni* cell counts decreased dramatically (Figure 1). Especially, the *C. jejuni* cell counts decreased rapidly at higher storage temperatures (Figure 1). Kim et al [27] presented that *C. jejuni* in beef tartare survived longer at low temperatures, because of *sodB*, *katA*, and *clpP* gene expression by *C. jejuni*. As *C. jejuni* had a longer survival time in the vacuum-packaged seasoned jerky (δ = 21.855) than in the aerobic-packaged seasoned jerky (δ = 1.352) at 10°C (data not shown), a primary model was developed for the vacuum-packaged seasoned jerky for the worst-case scenario. The R^2^ values of the primary model ranged from 0.870 to 0.961, indicating that the developed primary model was appropriate to describe the kinetic behavior of *C. jejuni* in jerky (Table 1). The δ values for *C. jejuni* in the vacuum-packaged seasoned jerky decreased from 21.855 to 0.159, as the temperature increased. To evaluate the effect of the temperature on δ, a secondary model was developed, and the equation was δ = (−9.3872)+(315.2666/T) with an R^2^ of 0.941 (Figure 2, Table 2). Validation of the model performance showed that the RMSE value was 0.447, and it indicates that the developed models were appropriate to predict the *C. jejuni* cell counts in jerky during storage and distribution.

### Storage time and temperature

The time and temperature for market storage were collected by personal communication at the market. On average, the jerky was sold within one month and kept at room temperature. Thus, an appropriate probabilistic distribution for the time and temperature in the market was the Pert distribution with parameters (0, 720, 2,160) and (0, 20, 25), respectively (Table 2). The transportation time from the market was at a minimum of 0.325 h and at a maximum of 1.643 h [24]. In addition, the minimum, mean, and maximum food temperatures during transportation were 10°C, 18°C, and 25°C, respectively [24]. The jerky was usually consumed within approximately 120 h at home and stored for up to 720 h at room temperature. Thus, the appropriate distribution for the time and temperature at home was the Pert distribution with parameters (0, 120, 720) and (15, 20, 25), respectively (Table 2).

### Jerky consumption in Korea

**Figure 3:**
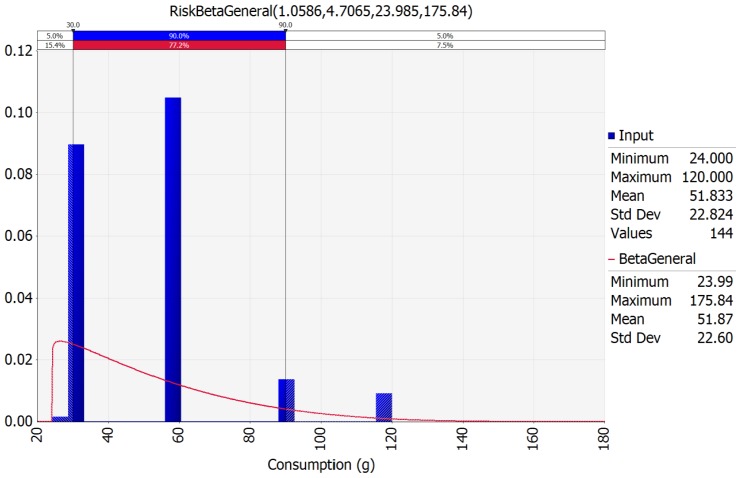
BetaGeneral probabilistic distribution for the jerky intake amount fit with @RISK 6.0.

As a result of fitting the consumption data with the @RISK program, a BetaGeneral distribution was optimal, and the daily consumption was 51.83 g on average per person (Figure 3). According to a survey by MFDS [25], the daily consumption frequency was 0.61%. This means that most people do not consume jerky, and they only consume a small amount compared to the increase in total meat intake.

### Dose-response model and risk characterization

To estimate the probability of foodborne illness from the consumption of *C. jejuni* cells, the following dose-response model [26] was utilized, because it is the most commonly used and was recently developed.

$$P_{inf}(n)=1-{\left(1-p_{1}\right)}^{n}$$

Where *p*_1_ is the probability of consuming *Campylobacter* cells, which is the value described by the Beta distribution [RiskBeta (0.145, 7.59)], *n* is the number of consumed *Campylobacter* cells, and *P_inf_* is the probability of infection. To estimate the probability of illness from jerky per person per day, the *P_inf_* (*n*) values were multiplied by *P_ill_*_|_*_inf_* (fixed 0.33) as follows. *P_ill_*_|_*_inf_* is the probability of illness from an infection, which has been suggested by Nauta et al [28].

$$\text{Probability of illness per person per day}=P_{inf}(n)\times P_{ill|inf}$$

**Figure 4:**
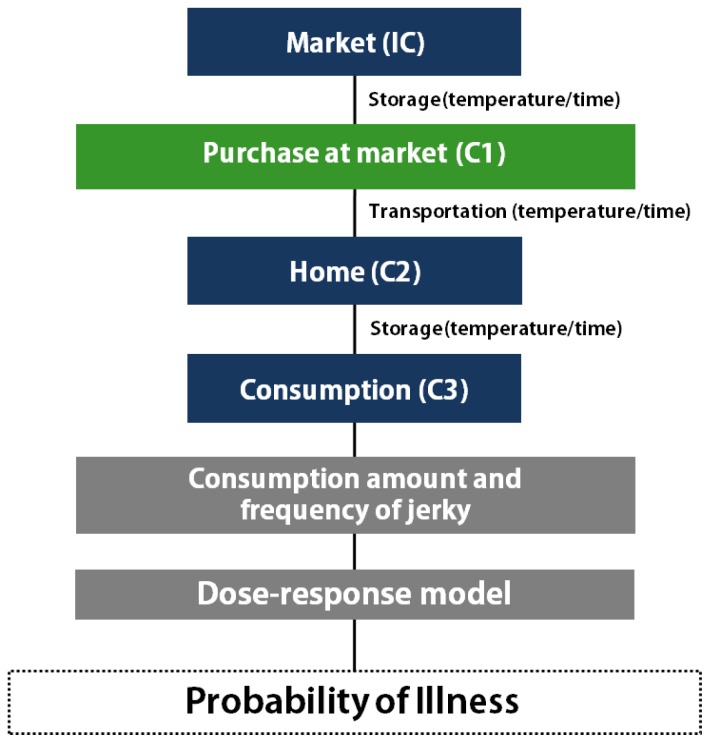
Scheme for the microbial risk assessment of *Campylobacter jejuni* in jerky.

**Figure 5:**
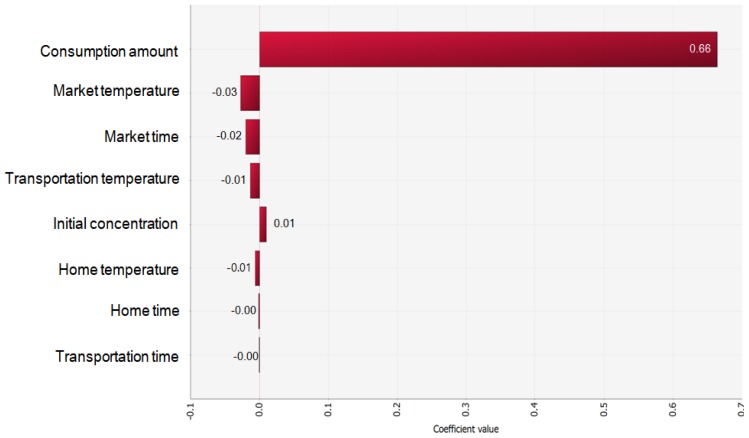
The correlation coefficient values of the risk factors that affect the probability of illness caused by jerky consumption per person per day.

With all the above data, the simulation model was prepared with a scenario for consumption at home (Figure 4). As the consumption frequency was not high, the risk was assessed by either including or excluding the consumption frequency. The risk estimated with the frequency indicates the overall risk in Korea, and the risk estimated without the frequency indicates the risk for a person who eats jerky. The probability of *C. jejuni* foodborne illness from jerky consumption per person per day was 1.56×10^−12^ with the consumption frequency, and 1.76×10^−8^ without the consumption frequency, showing the probability of illness per person per serving (Table 3). These results indicate that the risk of *C. jejuni* foodborne illness from jerky intake can be considered low in Korea. This result is similar to the risk of *Campylobacter* spp. infection from ham, and much lower than the risk from raw beef offal [29,30]. As a result of the sensitivity analysis, the most influential risk factor was the consumption amount. Additionally, the market temperature, time, transportation temperature, and risk were negative correlations (Figure 5), indicating that the *C. jejuni* counts decrease during distribution.

In conclusion, the quantitative risk assessment suggests that the risk of *C. jejuni* infection from the intake of jerky is low in Korea. Even if the jerky is contaminated with *C. jejuni* at a factory, the pathogen is destroyed during the distribution and storage.

## Figures and Tables

**Table 1:** δ and ρ calculated by the Weibull model [22] for Campylobacter jejuni in vacuum packaged seasoning jerky during storage at 10°C, 20°C, 25°C, and 30°C

Kinetic parameters	Temperature (°C)			

	10	20	25	30
δ (h)	21.855±4.773	6.973±1.853	3.407±0.774	0.159±0.036
ρ	0.484±0.078	0.468±0.051	0.557±0.060	0.273±0.002
R^2^	0.939	0.961	0.920	0.870

δ, required time for the first decimal reduction; ρ, shape of curve.

**Table 2:** Simulation model and formulas in Excel spreadsheet used to calculate the risk of *Campylobacter jejuni* in jerky with @RISK

Definition	Variable	Formula	Reference
Product			
Pathogens contamination level			
Initial contamination level (log CFU/g)	IC	= RiskUniform(−2,0.48)	
Market			
Market storage			
Storage time in market (h)	Mark-time_st_	= RiskPert(0,720,2160)	Personal communication
Storage temperature in market (°C)	Mark-temp_st_	= RiskPert(0,20,25)	Personal communication
Growth			
Treatment time for the first decimal reduction	Delta	= (−9.3872)+(315.2666/Mark-temp_st_)	This research
Shape	ρ	= Fixed 0.445588	This research
Contamination level before transportation (log CFU/g)	C1	= IC− (Mark-time_st_/Delta)^ρ^	[31]
Transportation			
Transportation time (market to home) (h)	Trans-time	= RiskPert(0.325,0.984,1.643)	[24]
Food temperature during transportation (°C)	Trans-temp	= RiskPert(10,18,25)	[24]
Growth			
Treatment time for the first decimal reduction	Delta	= (−9.3872)+(315.2666/Trans-temp)	This research
Shape	ρ	= Fixed 0.445588	This research
Contamination level before home (log CFU/g)	C2	= C1−(Trans-time/Delta)^ρ^	[31]
Home			
Home storage			
Storage time until consumption (h)	Home-time_st_	= RiskPert(0,120,720)	This research
Food temperature until consumption (°C)	Home-temp_st_	= RiskPert(15,20,25)	This research
Growth			
Treatment time for the first decimal reduction	Delta	= (−9.3872)+(315.2666/Home-temp_st_)	This research
Shape	ρ	= Fixed 0.445588	This research
Contamination level before consumption (log CFU/g)	C3	= C2− (Home-time_st_/Delta)^ρ^	[31]
Consumption			
Daily consumption average amount (g)	Consump	= RiskBetaGeneral (1.0586,4.7065,23.985,175.84)	[25]
Daily consumption frequency (%)	ConFre	Fixed 0.60971	[25]
Daily non consumption frequency (rate)	CF(0)	= 1–0.60971/100	[25]
Daily consumption frequency (rate)	CF(1)	= 0.60971/100	[25]
Distribution for consumption frequency	CF	= RiskDiscrete({0,1},{CF(0), CF(1)})	[25]
Daily consumption average amount considered frequency	Amount	= IF(CF=0,0,Consump)	[25]
Dose-response			
*Campylobacter* amount	n	= 10^C3^×Amount	
Parameter	α	Fixed 0.145	[26]
Parameter	β	Fixed 7.59	[26]
Dose-response distribution	P1	= RiskBeta(α, β)	[26]
Risk			
Probability of infection due to ingestion of n	P_inf_(n)	= 1−(1–P1)^n^	[28]
Probability of illness given infection	P_ill│inf_	Fixed 0.33	[28]
Probability of illness/person/d	Risk	= P_inf_(n)×P_ill│inf_	[28]

CFU, colony-forming unit.

**Table 3:** Probability of foodborne illness of *Campylobacter jejuni* per person per day and per person per serving by consumption of jerky

Items	5%	25%	50%	95%	99%	Mean
Probability of illness/person/d	0	0	0	0	0	1.56×10^−12^
Probability of illness/person/serving	0	0	0	1.30×10^−10^	1.11×10^−8^	1.76×10^−8^
